# Association between serum uric acid and metabolic syndrome components in prepubertal obese children (Tanner Stage I) from Nuevo León, Mexico - a preliminary study

**DOI:** 10.1186/s40608-017-0160-6

**Published:** 2017-07-03

**Authors:** Elizabeth Solis Perez, Mario Alberto González Medina, Manuel Lopez-Cabanillas Lomeli, Verónica Tijerina González, Jesús Zacarías Villarreal Pérez, Fernando J. Lavalle González, Victorine Imrhan, Shanil Juma, Parakat Vijayagopal, Kittipong Boonme, Chandan Prasad

**Affiliations:** 1 TWU-UANL Consortium to Promote Research in Obesity and Obesity-related Diseases, Monterrey, Mexico; 2 School of Public Health and Nutrition of Universidad Autónoma de Nuevo León, Monterrey, Mexico; 3 Endocrinology Service UANL Hospital Dr. José Eleuterio González, Monterrey, NL Mexico; 4 Department of Nutrition and Food Sciences, Texas Woman’s University, PO Box 425888, Denton, TX 762014-5888 USA; 5 School of Management, Texas Woman’s University, Denton, TX USA; 6 Sectional of Endocrinology, Department of Medicine, LSU Health Sciences Center, New Orleans, LA USA

## Abstract

**Background:**

Metabolic syndrome (MetS) is a major risk factor for cardiovascular disease and diabetes. Previous studies in obese children demonstrating a positive association between serum uric acid (sUA) and components of MetS are confounded by lack of uniformity in age and pubertal status of children. Therefore, we have examined the role of sUA in MetS and its components in pre-pubertal children (Tanner Stage I, age ≤ 9 years).

**Methods:**

Pre-pubertal obese children (32 boys, 27 girls, age 6–9 years) were recruited from Nuevo Leon, Mexico. For comparison, an equal number of children with normal body mass index (BMI) in the same age range (22 Boys, 39 girls, age 6–9 years) were also recruited from the same community. Presence of MetS and its components was defined according to the criteria of International Diabetes Federation. Fasting blood was analyzed for lipids, glucose, insulin, and uric acid.

**Results:**

Among the obese children, sUA was positively associated with insulin resistance and hypertriglyceridemia and negatively associated with high density lipoprotein-cholesterol (HDLc). Subjects were three times more likely to have a MetS diagnosis per one unit (md/dL) difference in sUA. Of the 59 obese pre-pubertal children, 20 were classified as having MetS defined by the presence of abdominal obesity and two or more of other components described under methods. Of these, 57.1% (20/61) had sUA between 5.1 and 7.1 mg/dl.

**Conclusions:**

The findings of this study clearly indicate a positive relationship between uric acid and MetS and its components in pre-pubertal obese children with Tanner stage I and ≤9 years of age.

## Background

There has been a precipitous rise in the prevalence and magnitude of childhood obesity over the last few decades [1]. Unfortunately, it is difficult to determine the rate of prevalence of metabolic syndrome (MetS) in obese children due to complexity of definition, and differences such as ethnicity, gender and sexual maturity [2]. However, most studies support the notion that the prevalence of MetS is high among obese children, and it increases with degree of obesity [2].

Similar to adult population, there have been scores of epidemiologic and observational studies examining the role of serum uric acid (sUA) in MetS in children [3–11]. While in general results of these studies support a direct relationship between sUA and MetS, the data for analyses were pooled from pre-, peri- and post-pubertal children without any control for differences in their sexual maturity [3–11]. While the above studies [3–11] support the conclusion that the odds ratio of having metS or one or more its components is associated with sUA, seven of the studies [3, 4, 6, 7, 9–11] include data from pre-pubertal, post-pubrtal and post-pubertal children ranging in age between 4 and 18 years making it difficult to assess the role of sUA in MetS in just pre-pubertal children. The remaining two studies [5, 8] included peri-pubertal children ranging in age between 10 and 13 years.

Since sex steroids are known to control uricemia as well as sexual maturity [12–15], the lack of regard for sexual maturity in subject selection in these studies requires a re-examination of the relationship. Therefore, in the present study, we have examined the role of sUA in MetS and its components in obese pre-pubertal (Tanner stage I) elementary school children from Mexico and compared it with sexual maturity matched normal body mass index (BMI) children in the same age range.

## Methods

### Study population

In this cross-sectional study, pre-pubertal children were recruited through a summer health camp for childhood obesity prevention at Universidad Autónoma de Nuevo León [16]. For comparison, children with normal BMI were also recruited from the same community. The study subjects were from elementary schools in metropolitan area of Monterrey City and rural municipalities of Nuevo León, México. The summer camps are organized annually for obese children who come from low-income families living in poverty or extreme poverty and qualify for medical care through the public health system of México. The same socio-economic measures were applied to select the children with normal BMI.

### Subject selection

The study was approved by the Research Ethical Committee of the Public Health and Nutrition School at Universidad Autónoma de Nuevo León, which is registered with the State Research Ethical Committee in concordance with the Health General Law of Mexico. All the children and their parents signed a letter of agreement and consent form.

The major goal of this study was to examine the association between sUA with MetS and its components in pre-pubertal children. Both sUA and the onset of puberty are affected by gender, age and adiposity [17]. Therefore, it was important that children selected for this study be not only comparable in age, but are also pre-pubertal. To this end, a pediatrician screened 200 consecutive obese children in the age group of 6–9 years for sexual maturity (Tanner Stages of puberty). Of these, 59 obese children were classified as Tanner Stage I (Table 1). A similar screening of normal BMI children yielded 61 children belonging to Tanner Stage I [16].

**Table 1:** Characteristics of the study population

Variables	Normal (*n* = 61)	Obese (*n* = 59)	*p*-value
Age, years	7.3 ± 1.1	8.0 ± 1.0	0.001^a^
Tanner Stage	I	I	
Body Mass Index	16.2 ± 1.2	26.2 ± 4.4	0.000^a^
Waist circumference, cm	55.8 ± 3.9	83.8 ± 11.0	0.000^b^
Systolic blood pressure, mmHg	83.0 ± 8.9	94.1 ± 10.9	0.000^a^
Diastolic blood pressure, mmHg	59.6 ± 7.1	63.5 ± 9.6	0.056^a^
Fasting glucose, mg/dL	80.6 ± 6.6	80.2 ± 6.7	0.741^b^
Fasting Insulin, UI/mL	8.1 ± 3.2	26.8 ± 20.1	0.000 ^a^
HOMA	1.6 ± 0.7	5.3 ± 4.0	0.000^a^
Triglycerides, mg/dL	104.5 ± 31.1	155.4 ± 88.9	0.001^a^
Total cholesterol, mg/dL	160.7 ± 25.3	163.6 ± 34.0	0.598^b^
LDL-C, mg/dL	87.3 ± 22.4	93.8 ± 28.5	0.165^b^
HDL-C, mg/dL	52.9 ± 13.4	39.0 ± 8.2	0.000^a^
Uric Acid, mg/dL	3.4 ± 0.6	4.4 ± 0.9	0.000^b^

All data except Tanner Stage are presented as mean ± SD. ^a^The Mann-Whitney U test. ^b^t-Test for independent samples

### Anthropometric and blood pressure measurement

Weight was measured using digital scales (TanitaBC-533) while subjects were minimally clothed and without shoes, recorded to the nearest 100 g. Height was measured to the nearest 1 cm using a non-elastic tape meter while subjects were in a barefoot standing position, with their shoulders in a normal position. BMI was calculated as weight in kilograms divided by the square of height in meters. Presence of obesity was determined by BMI-for-age using WHO Reference [18]. Blood pressure (BP) was measured twice in the right arm of subjects who had been resting for at least 10 min in a seated position using a mercury sphygmomanometer.

### Metabolic syndrome and its components

Presence of MetS was determined using the definition of the International Diabetes Federation (IDF) [19]. According to the IDF definition, someone has the metabolic syndrome if he or she has central adiposity (waist circumference (WC) ≥ 90th percentile) plus two or more of the following four factors [19]: a) systolic blood pressure ≥ 130 mmHg or diastolic blood pressure ≥ 85 mmHg, b) fasting triglycerides (TG) ≥150 mg/dL, c) high density lipoprotein (HDLc) < 40 mg/dL and d) fasting glucose ≥100 mg/dL).

### Biochemical measurements

Blood was collected between 07:30 and 08:00 AM from the antecubital vein after an 8–12 h overnight fast and centrifuged within 2 h for separation of serum. Aliquoted samples were stored at −20 °C until analyses. Serum Total cholesterol and TG were determined enzymatically by an autoanalyzer using commercial kits available (Beckman Coulter, Inc., CA, USA). Serum HDLc was measured similarly after precipitation with magnesium phosphotungstate. Serum low density lipoprotein-cholesterol (LDLc) was calculated using Friedwald’s formula [20] as shown below.

$$ \left[ LDL- chol\right]=\left[ Total\  chol\right]-\left[ HDL- chol\right]-\left(\left[ TG\right]/5\right)\  where\  all\  concentrations\  are\  given\  in\  mg/ dL $$

Fasting plasma glucose was measured via colorimetric assay and insulin levels were determined using radioimmunoassay. Serum uric acid levels were determined colorimetrically using Uricase. Assays were done in triplicate and were performed at the General and Endocrinology Laboratories of the Hospital Universitario Dr. José Eleuterio González. The laboratory routinely monitors both inter- and intra-assay coefficients of variation for all assays with a goal to keep it at 5% or below. For example, in our insulin assay, inter- and intra-assay coefficient of variation was 2.9–3.8% and 2.5–4.4%, respectively. Insulin-resistance (IR) was evaluated with the aid of homeostasis model assessment (HOMA) and defined as HOMA >2.7 (HOMA-IR) [21].

### Statistical analyses

Continuous data is presented as means ± SD. A post-hoc power analysis was performed to determine the statistical power to detect significant differences for the main comparison. Using a moderate effect size of 0.50, an alpha of 0.05, and a combined sample size *n* = 120; we calculated a power of 0.845, that meets the minimum suggested power for a study [22]. Data were analyzed using multivariate statistical software SPSS (version 22). Differences in the components of the MetS, age, Tanner stage, fasting insulin and HOMA among the different groups were analyzed by descriptive and exploratory statistical analyses. Mann-Whitney U test was applied to examine statistical significance among the variables such as age, BMI, systolic pressure, diastolic pressure, fasting insulin, HOMA, triglycerides and HDLc. A t-test for independent samples was used to examine differences in means between the obesity group and normal BMI control group based on identified factors: glucose, waist circumference, total cholesterol, and LDLc. To determine the statistical association between the presence of Met S and sUA, a binary logistic regression equation was utilized. The predictive variables were age, (continuous), the concentration of sUA (continuous), gender (dichotomous) and BMI (continuous). The scatter-plots were made with their respective linear correlation and equation of simple linear regression to examine the relationship between concentration of sUA to fasting insulin, HOMA, HDLc, and triglycerides.

## Results

**Fig. 1:**
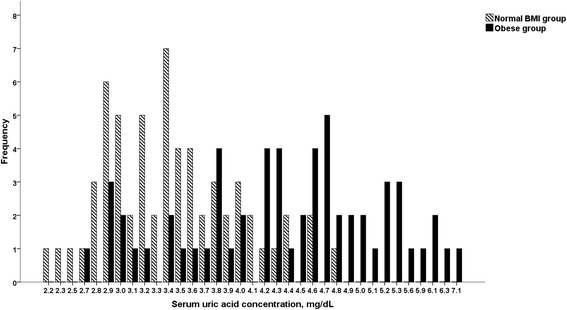
Distribution of the serum uric acid in obese and normal BMI pre-pubertal children (*n* = 120) aged 6–9 years. Frequency = Number of children

Distribution of sUA in obese and normal BMI pre-pubertal children in the age group 6–9 years is shown in Fig. 1. There were more children with high sUA in the obese group (43/59, Range: 2.5–7.5 mg/ml; 70% with sUA ≥4.0 mg/ml) than in the normal BMI group (9/61, Range: 2.2–4.8 mg/ml; 15% with sUA ≥4.0 mg/dl) (*p* = 0.001).

The baseline characteristics of children in the obese and normal BMI groups are shown in Table 1. Both normal BMI and obese groups were matched closely in sample size and age. Children in the obese group were only 8 months older, however, children in both groups were under 9 years of age. Furthermore, all obese and normal BMI children were pre-pubertal with Tanner stage I. Children in the obese group had significantly greater waist circumference (*p* = 0.001), increased systolic blood pressure (*p* = 0.001), elevated fasting insulin (*p* = 0.001), insulin resistance measured by HOMA (*p* = 0.001), higher TG (*p* = 0.002), higher sUA (*p* = 0.001), and lower HDLc (*p* = 0.001) compared to the normal BMI group. These data clearly show the presence of components of MetS in pre-pubertal obese children.

Of the 59 obese pre-pubertal children, 32 were boys and 27 were girls. We next analyzed gender differences between the prevalence of the components of MetS in obese children. Results presented in Table 2 show that obese girls were at a higher risk for insulin resistance than obese boys as shown by increased fasting insulin (*p* = 0.003) and HOMA values (*p* = 0.00). Also, obese girls had significantly lower HDLc than obese boys (*p* = 0.014). Interestingly, however, there was no difference in the levels of sUA between obese boys and obese girls (*p* = 0.543).

**Table 2:** Characteristics of the obese group by gender

	Obese		
Variables	Boys (*n* = 32)	Girls (*n* = 27)	*p*-value
Age, years	7.8 ± 1.0	8.2 ± 0.9	0.132^a^
Tanner Stage	I	I	
Body Mass Index	26.3 ± 4.8	26.2 ± 3.9	0.999^a^
Waist circumference, cm	84.4 ± 12.2	83.0 ± 9.5	0.646^b^
Systolic blood pressure, mmHg	95.0 ± 13.1	92.9 ± 7.7	0.734^a^
Diastolic blood pressure, mmHg	65.6 ± 11.3	61.1 ± 6.4	0.122^a^
Fasting glucose, mg/dL	80.4 ± 6.4	79.9 ± 7.2	0.761^b^
Fasting Insulin, UI/mL	20.5 ± 13.5	34.2 ± 24.1	0.002^a^
HOMA	4.1 ± 2.7	6.7 ± 4.7	0.003^a^
Triglyceride, mg/dl	144.3 ± 88.7	168.4 ± 89.0	0.191^a^
Total cholesterol, mg/dl	167.5 ± 32.9	158.9 ± 35.4	0.337^b^
LDL-C, mg/dL	98.0 ± 29.4	88.9 ± 27.2	0.226^b^
HDL-C, mg/dL	41.3 ± 8.5	36.2 ± 7.0	0.017^b^
Uric Acid, mg/dL	4.5 ± 1.0	4.3 ± 0.7	0.430^b^

^a^The Mann-Whitney U test. ^b^t-Test for independent samples

We used a multivariate analysis to establish the association between sUA and components of MetS after adjusting for gender, age, and BMI (Table 3). The results show that with one unit difference (1 unit = 1 mg/dL) in sUA, there were 3.9 times more likely to have a MetS diagnosis as defined by IDF. Similarly, higher sUA levels are significantly associated with high waist circumference, high TG and low HDLc. Of the 59 obese pre-pubertal children, 20 were classified as having MetS defined by the presence of abdominal obesity and two or more of other components described under methods. Of these, 57.1% (20/61) had sUA between 5.1 and 7.1 mg/dl.

**Table 3:** Adjusted odds ratios (95% CI)* for association 1 between the MetS and 2 its components with sUA

	Variables	Odds ratio	95% CI	*p*-value
Metabolic syndrome *n* = 59	Age (yr)	0.620	[0.302, 1.273]	0.193
	Uric Acid (mg/dL)	3.942	[1.589, 9.775]	0.003
	Gender	0.208	[0.048, 0.901]	0.208
	BMI (kg/m2)	1.113	[0.949, 1.304]	0.187
Abdominal obesity (waist circumference > 90th percentile) *n* = 53	Age (yr)	0.677	[0.328, 1.397]	0.291
	Uric Acid (mg/dL)	3.854	[1.518, 9.787]	0.005
	Gender	0.212	[0.048, 0.930]	0.040
	BMI (kg/m2)	1.074	[0.907, 1.271]	0.410
High triglyceride (≥150 mg/dL) *n* = 25	Age (yr)	0.504	[0.124, 2.048]	0.338
	Uric Acid (mg/dL)	2.267	[0.560, 9.180]	0.251
	Gender	0.474	[0.029, 7.801]	0.601
	BMI (kg/m2)	1.406	[0.835, 2.369]	0.200
Low HDL-C (<40 mg/dL) *n* = 33	Age (yr)	0.210	[0.031, 1.008]	0.110
	Uric Acid (mg/dL)	65.751	[2.509,1723.076]	0.012
	Gender	0.040	[0.001, 2.003]	0.107
	BMI (kg/m2)	1.559	[0.764, 3.182]	0.223

All the models were adjusted by gender (men compared with women), age (continuous), body mass index (continuous) and concentration of uric acid (continuous)

*The unit of the exposures for the OR estimate was the presence or absence of Metabolic

Syndrome or its components- abdominal obesity, high triglycerides and low HDL-C

**Fig. 2:**
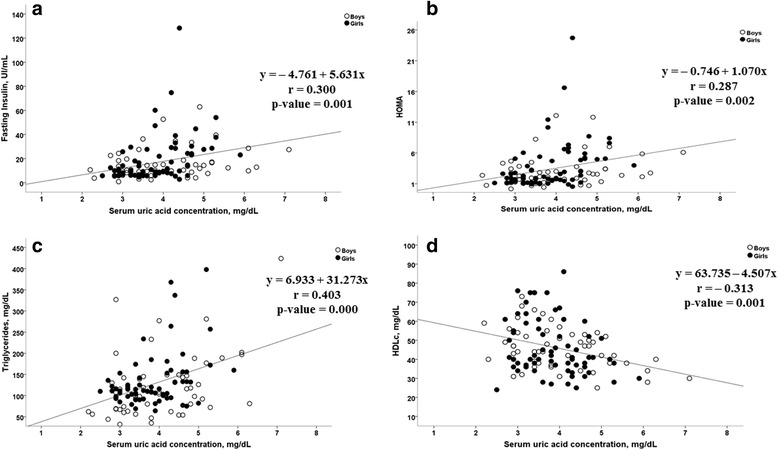
Association between serum serum uric acid concentration and fasting insulin, HOMA, trigycerides and HDLc. **a** Insulin (y = − 4.761 + 5.631×; *r* = 0.287; *p*-value = 0.001, two-tailed analysis; *n* = 119). **b** HOMA (y = − 0.746 + 1.070×; *r* = 0.300; *p*-value = 0.002, two-tailed analysis; *n* = 119). **c** Triglycerides (y = 6.933 + 31.273×; *r* = 0.403; *p*-value = 0.000, two-tailed analysis; *n* = 119). **d** HDLc (y = 63.735–4.507×; *r* = − 0.313; *p*-value = 0.001, two-tailed analysis; *n* = 119)

Data presented in Fig. 2 show the positive relationship (*p* < 0.05) between elevated sUA and measures of insulin sensitivity (insulin in Panel A and HOMA in Panel B). Similarly, hyperuricemia was positively associated (*p* < 0.05) with hypertriglyceridemia in Panel C and and negatively associated with HDLc in Panel D.

## Discussion

The conclusion of the earlier studies examining relationship between sUA and higher odds ratio for MetS or its components in children are similar to our observation [3–11]. However, the conclusions reached in these studies were confounded because they did not control for race/ethnicity, gender, age and stages of sexual maturity in subject selection [3–11]. For example, Gill-Gampos et al. reported a positive association between the features of insulin resistance and hyperuricemia in prepubertal children ranging in ages between 6 and 12 years [23]. Similar to our results, Viazzi et al. reported that hyperuricemia was associated with increased blood pressure in children ranging in age between 6 and 18 years [24]. Multiple other studies have examined such associations and have suggested a role for hyperuricemia in MetS and its components in younger population [3–11]. The data presented in Table 3 are the first to show an association between risks for known components of MetS and sUA in obese prepubertal children (age: 6–9 years and Tanner Stage I) in Monterrey, Mexico. These data offer the possibility of use of sUA as a predictor of MetS in pre-pubertal children.

The results of these studies offer only a predictive relationship between sUA and MetS in obesity. The cause and effect relationship between sUA and obesity can only be speculated at this time. Approximately two thirds of total body uric acid is produced endogenously, while the remaining one third results from metabolism of dietary purines [25]. Hyperuricemia may occur because of increased production (overproducers), decreased excretion (underexcretors), or a combination of these two mechanisms. In a recent study, Tsushima et al. [26], demonstrated elevated uric acid secretion from whole adipose tissue in obese vs. lean mice, and from 3 T3-L1 adipocytes under hypoxia suggesting that purine catabolism to uric acid in adipose tissue could be enhanced in obesity. In support of decreased excretion, Yamashita et al. [27] reported marked reduction of renal uric acid excretion in obese subjects and its improvement by a low-calorie diet. Matsuura et al. [28] observed that while all obese subjects had higher sUA than normal-weight control subjects, subjects with visceral obesity were linked more closely to overproduction and under excretion of uric acid. Taken together, these studies support the thesis that both uric acid production and excretion play an active role in determining the state of sUA. However, since these foregoing observations came from animal, cell culture and adult human studies, their relevance to prepubertal children needs to be examined.

### Limitations of this study

While the strength of this study lies in our demonstration of an association between higher sUA and higher odds ratio for components of MetS, this is a cross-sectional study and causality cannot be inferred. The relationship between pediatric obesity and MetS is complex because the risk for sequelae of both obesity and MetS vary among individuals based on ethnicity, socio-economic status and associated lifestyle practices [29].

## Conclusions

Compared with normal weight children, obese children are more likely to experience hyperuricemia. Also there was a positive relationship between sUA and MetS and its components in pre-pubertal obese children with Tanner stage I and ≤9 years of age. Further studies are needed to understand role of uric acid in eliciting MetS and its components these children.

## Abbreviations

BMI: Body mass index
BP: Blood pressure
HDLc: High density lipoprotein-cholesterol
HOMA: Homeostasis model assessment
IR: Insulin-resistance
LDLc: Low density lipoprotein-cholesterol
MetS: Metabolic syndrome
OR: Odds ratio
SD: Standard deviation
sUA: Serum uric acid
TG: Triglycerides
WC: Waist circumference
